# The meiosis-specific modification of mammalian telomeres

**DOI:** 10.4161/cc.29350

**Published:** 2014-05-28

**Authors:** Hiroki Shibuya, Yoshinori Watanabe

**Affiliations:** 1 Laboratory of Chromosome Dynamics; Institute of Molecular and Cellular Biosciences; University of Tokyo; Tokyo, Japan; 2 Graduate School of Agricultural and Life Science; University of Tokyo; Tokyo, Japan

**Keywords:** meiosis, chromosome, telomere, nuclear envelope, cohesin

## Abstract

During meiosis, rapid chromosome movements within the nucleus enable homologous chromosomes to acquire physical juxtaposition. In most organisms, chromosome ends, telomeres, tethered to the transmembrane LINC-complex mediate this movement by transmitting cytoskeletal forces to the chromosomes. While the majority of molecular studies have been performed using lower eukaryotes as model systems, recent studies have identified mammalian meiotic telomere regulators, including the LINC-complex SUN1/KASH5 and the meiosis-specific telomere binding protein TERB1. This review highlights the molecular regulations of mammalian meiotic telomeres in comparison with other model systems and discusses some future perspectives.

## Introduction

Meiosis is a specialized cell division for gametogenesis comprising 2 rounds of cell division, meiosis I and II, after a round of DNA synthesis. During meiosis I, homologous chromosomes acquire physical connections by homolog pairing, synapsis, and reciprocal recombination. The physical connection between homologs is not only important for generating genetic variation by exchanging genetic materials between paternal and maternal chromosomes, but, more crucially, is required for correct homolog disjunction in the following chromosome segregation during metaphase I.

In 2006, Hiraoka and colleges presented an extensive characterization of the meiotic telomere apparatus of fission yeast, *Schizosaccharomyces pombe*.^1^ They identified the meiosis-specific protein, Bqt1/2, that associates with meiotic telomeres by binding to the conserved telomere binding protein, Rap1, and the accumulation of the transmembrane LINC complex (linker of nucleoskeleton and cytoskeleton) to the telomere association sites on the nuclear envelope (NE). In fission yeast meiosis, the LINC complex comprises the inner nuclear membrane SUN domain protein, Sad1, and the outer nuclear membrane KASH domain protein, Kms1/2, and acts as a molecular linker between telomeres and microtubule-dependent cytoskeletal motors, such as the Dynein–Dynactin complex and Kinesins,^1^^,^^2^ enabling chromosome movement along the NE. This movement is thought to facilitate homolog juxtaposition and subsequent reciprocal recombination.

Similar regulation is also found in budding yeast, *Saccharomyces cerevisiae*. In this organism, the meiosis-specific telomere binding protein Ndj1 associates with meiotic telomeres, leading to the accumulation of the transmembrane LINC complex, Mps3, and Csm4, facilitating actin-dependent chromosome movements along the NE.^3^^-^^6^

Also in worm, *Caenorhabditis elegans*, specific chromosomal parts other than telomeres, called pairing centers, recruit a set of zinc-finger proteins, HIM-8 and ZIM proteins, that accumulate the LINC-complex, SUN-1, and ZYG-12 on the NE, facilitating Dynein motor-dependent chromosome movements.^7^^-^^9^

These findings have suggested the evolutionally conserved aspects of meiotic chromosome movements driven by cytoskeletal forces transmitted by the LINC complex to specific chromosomal parts (usually telomeres, but, exceptionally, pairing centers, as in worms) and the requirement for proper pairing/synapsis and recombination of homologous chromosomes during meiotic prophase I (Fig. 1; Table 1).

**Figure:**
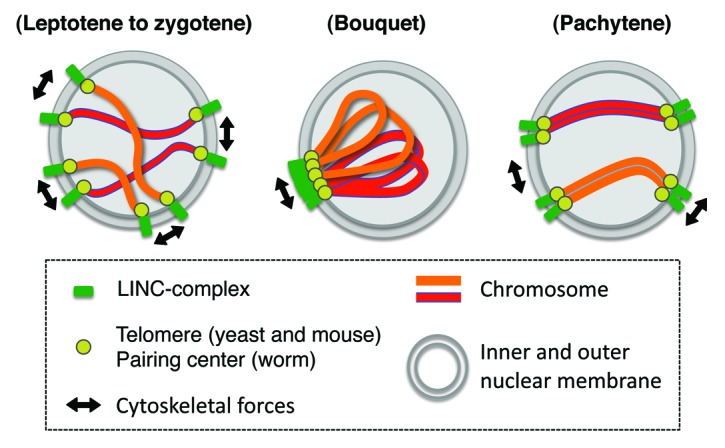
**Figure 1. ** The conserved rapid chromosome movement during meiotic prophase I. During meiosis, telomeres (or pairing center in worm) are tethered to the NE and assemble the transmembrane LINC complex to the association sites. LINC complex, associating with cytoskeletal motors, facilitates the rapid chromosome movements along the NE (leptotene to zygotene), accompanying transient bouquet configuration of meiotic chromosomes (bouquet). Then, chromosome acquires the homolog association (pachytene).

**Table:** **Table 1.** Responsible factors regulating the meiosis-specific rapid chromosome movements in various model systems

	Fission yeast	Budding yeast	Worm	Mouse
**SUN domain protein (inner nucler membrane)**	Sad1	Mps3	SUN-1	SUN1
**KASH domain protein (outer nuclear membrane)**	Kms1, 2	Csm4	ZYG-12	KASH5
**Chromosomal scaffold**	Bqt1/2	Ndj1	HIM8, ZIM-1,2,3	TERB1
**Motor**	Dynein, Dynactin, Kinesin	Actin motors	Dynein	Dynein, Dynactin

Cytoplasmic motor forces, generated by MT-dependent motors (fission yeast, worm, and mouse) or actin motors (budding yeast), are transmitted to the specific chromosomal parts, generally telomeres (fission yeast, budding yeast, and mouse), or, exceptionally, pairing centers (worm), through the associations between meiosis-specific chromosomal scaffold proteins and transmembrane LINC complex, composed of SUN domain protein, locating in the inner nuclear membrane, and KASH domain protein, locating in the outer nuclear membrane.

## Identification of a Mammalian LINC Complex Required for Meiotic Homolog Juxtaposition

Among the 6 SUN domain protein paralogs identified in mammals, Min and colleagues found that one of the ubiquitously expressed SUN domain proteins, SUN1, plays an essential role in the progression of mouse meiosis.^10^ They demonstrated that SUN1 usually localizes along the whole inner nuclear membrane, but accumulates at meiotic telomeres. They also showed that *Sun1* gene knockout mice are infertile due to defective homolog pairing/synapsis during meiosis.

Later, a novel meiosis-specific KASH domain protein, KASH5, was identified by 2 groups.^11^^,^^12^ KASH5 forms a complex with SUN1 and localizes to meiotic telomeres in a SUN1-dependent manner.^11^^,^^12^ Moreover, KASH5 associates with and localizes the cytoplasmic Dynein–Dynactin complex to meiotic telomeres. Phenotype analysis of *Kash5-KO* mice revealed that KASH5 is also essential for homologous chromosome pairing/synapsis and fertility.^12^ In a recent study, rapid telomere movements along the NE in live murine spermatocytes were filmed by tracing the signals of the GFP-fused telomere binding protein TRF1 (GFP-TRF1).^11^ This chromosome movement was almost completely lost for both spermatocytes from *Sun1-KO* and wild-type spermatocytes cultured in the presence of the MT-depolymerizing drug, nocodazole.^11^^,^^13^ These findings suggest that in mammals as well, meiotic chromosome movement along the NE is mediated by microtubule-dependent cytoplasmic motor forces, which are transmitted through the LINC complex, SUN1/KASH5 (Fig. 1; Table 1).

## Identification of a Meiosis-Specific Telomere Binding Protein, TERB1, in Mammals

While the LINC-complex is highly conserved among eukaryotes, the regulators of the telomere side are completely different even between fission and budding yeasts.^14^ Further, although the conserved telomere binding protein Rap1 acts as a scaffold for meiotic telomere proteins in both fission and budding yeasts, mutant mice lacking the expression of the RAP1 protein, the homolog of yeast Rap1, showed normal meiotic progression and fertility, leaving the regulator of mammalian meiotic telomeres elusive until recently.^15^

A recent study demonstrated localization-based screening for uncharacterized meiotically upregulated genes by in vivo electroporation of live mouse testes and identified a novel meiosis-specific protein, CCDC79/TERB1 (telomere repeat binding bouquet formation protein 1), as a meiotic telomere regulator conserved among vertebrates.^13^ *Terb1* mRNA expression is restricted to gonadal tissues in mouse, and immunolocalization revealed that TERB1 localizes to meiotic telomeres in both spermatocytes and oocytes throughout meiotic prophase I.

TERB1 has a TRF family protein-like, SANT/MYB-like DNA binding domain on its C terminus, suggesting that TERB1 might be evolutionally derived from TRF family proteins (Fig. 2A). Further, in vitro biochemical assays suggested that TERB1 forms a 1:1 stoichiometric heterocomplex with TRF1 through the TRFB (TRF1 binding) domain of TERB1, and that this heterocomplex formation is necessary and sufficient for TERB1 telomere localization in vivo. Conversely, TERB1 binds to the TRFH (TRF homology) domain of TRF1, a domain required for TRF1 homodimerization^16^ (Fig. 2A). It is proposed that TERB1 binds competitively to the TRFH domain of TRF1 to form a stoichiometric heterocomplex (or heterodimer) rather than a canonical TRF1 homodimer to modify meiotic telomeres as the machinery for chromosome movement (Fig. 2b).

**Figure:**
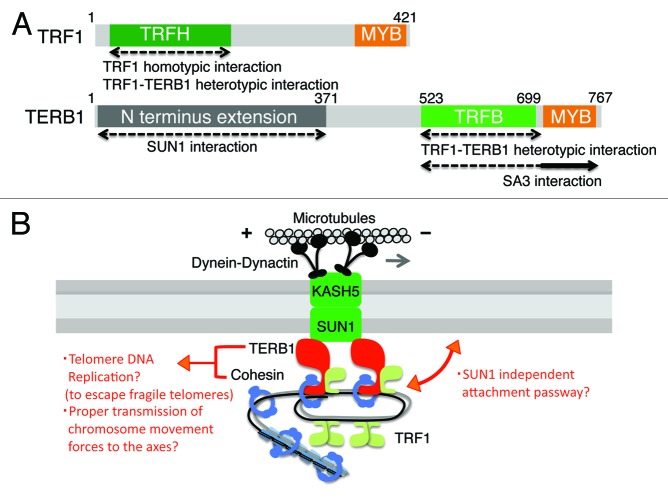
**Figure 2.** Modification of mammalian meiotic telomeres by TERB1. (**A**) Domain conformations of TRF1 and TERB1. TRF1 is composed of an N-terminus TRF homology domain, required both for TRF1 homodimerization and TERB1 hetero‐binding, and C-terminus MYB domain, required for telomere DNA binding. TERB1 is composed of N-terminus extension, which binds to SUN1’s N terminus, C-terminus TRFB domain, required for TRF1 binding and TERB1 telomere localization in vivo, and MYB domain. SA3 binding is mediated by TERB1 C terminus. In particular, the very C-terminal MYB domain (thick line) is essential for cohesin telomere accumulation in vivo. (**B**) Models and future perspectives (highlighted in red word) of telomere regulations during mammalian meiosis.

## TERB1 Regulates the Accumulation of SUN1/KASH5 at Meiotic Telomeres

TERB1 binds directly to the LINC complex protein SUN1 through its conserved N-terminus extension, suggesting its involvement in prophase chromosome movements (Fig. 2A). TERB1 gene trap mice, which lack TERB1 expression (*Terb1-KO*), develop normally, but, as in the case of *Sun1-KO*, are completely infertile. A detailed analysis of meiocytes from *Terb1-KO* revealed that the telomeres are partly detached from the NE, and that SUN1/KASH5 accumulations at the telomeres are completely abolished, even though some telomeres are situated near the NE.

Live imaging of chromosome axes and telomeres in spermatocytes revealed that there are 2 superimposed types of chromosome movements, random telomere movements and unidirectional rotation of whole chromosomes. These movements are almost completely abolished in *Terb1-KO* spermatocytes, as in *Sun1-KO*, suggesting that telomere accumulation of SUN1 as regulated by TERB1 is essential for the overall movement of meiotic chromosomes. Accordingly, homolog pairing/synapsis and recombination are significantly impaired in spermatocytes and oocytes of *Terb1-KO*. These observations highlight the functional analogies between TERB1 and other meiotic telomere/pairing center proteins so far reported in other model systems^14^^,^^17^ (Table 1).

## TERB1 MYB Domain Acts for Cohesin Recruitment

Further detailed analysis revealed an unexpected function of TERB1 in meiotic telomere regulation. Yeast 2-hybrid screening identified a meiotic cohesin subunit, SA3,^18^^,^^19^ as a TERB1 binding protein. The meiotic cohesin complex forms a chromosome axis (or axial element) along the whole chromosome that extends to the telomeric regions at both ends of the axis.^13^^,^^20^ This cohesin extension to the meiotic telomeres is abolished in *Terb1-KO* spermatocytes.^13^

The TERB1 MYB domain associates with SA3 and plays a role in cohesion accumulation at meiotic telomeres in vivo (Fig. 2A). Since the MYB domain usually acts in nucleotide binding,^21^ the TERB1 MYB domain might also bind to telomere repeat DNA as a form of TRF1 heterocomplex. If this is the case, TERB1 may associate with SA3 cohesin through different surfaces, reminiscent of the case previously reported for c-Myb transcription factor.^22^

*Terb1-KO* spermatocytes show a “fragile telomere” in zygotene-like prophase I spermatocytes, consistent with the loss of meiotic cohesin localization from telomeres, as observed in *Smc1β-KO*.^20^ Fragile telomeres were originally characterized in mitotic metaphase condensed chromosomes after the depletion of TRF1 or the SA1 cohesin subunit,^23^^-^^26^ a mitotic counterpart of SA3 that binds directly to TRF1 and is then recruited to mitotic telomeres.^27^^,^^28^ The precise molecular character of this cytologically defined malstructure remains elusive, although defects in telomere repeat DNA replication might cause this aberration. It is known that G-rich telomere duplex DNA tends to form a secondary structure, the G-quadruplex, which challenges the progression of the replication fork.^29^^,^^30^ To overcome this problem, TRF1-SA1 may have a role in promoting replication fork progression on telomere repeat DNA.^25^^,^^28^

Considering the analogies and the absence of mitotic cohesin expression, including SA1, during meiosis,^31^^-^^33^ it is possible that TERB1 and the SA3-containing cohesin complex (also containing SMC1β subunits^31^^,^^32^) may act in the efficient replication of telomere repeat DNA during pre-meiotic S phase, as do TRF1 and SA1 during mitotic S phase.

## Conclusions and Perspectives

Recent studies have revealed the molecular regulation of mammalian meiotic telomeres in mice.^10^^-^^12^ Especially, characterizations of the meiosis-specific telomere regulator TERB1 have revealed that mammalian meiotic telomeres are fundamentally modified for chromosome movement, which accompanies modification of the core shelterin complex by the formation of a TRF1–TERB1 heterocomplex instead of the canonical TRF1 homodimer.^13^ TERB1 then acts as a regulatory scaffold for the LINC complex SUN1/KASH5 and the meiotic cohesin complex that contribute to the formation of the rigid telomere structure (Fig. 2B). The involvement of the cohesin complex in meiotic telomere regulations is an intriguing finding that has never been reported in other model systems.

## Cohesin/Axial Element, for Chromosome Movements?

Earlier electron microscopic observations found that there is an electron-dense structure called the telomere attachment plate at NE association sites in murine spermatocytes.^34^ Because the axial element extends to and is integrated into the structure, one might argue that the cohesin/axial element enriched at meiotic telomeres could play a role in the proper transmission of chromosome driving forces to the chromosome axes. Detailed observations of chromosome movements or telomere structures in mutant spermatocytes lacking meiotic cohesin or the MYB domain of TERB1 will address this hypothesis in the future.

## Attachment Plate Structure, Built by TERB1?

A recent study found that telomeres are partly detached from the NE in *Sun1-KO* spermatocytes;^10^ however, the telomere attachment plate is properly formed at telomeres near the nuclear periphery.^35^ Our cytological observations found that TERB1 accumulates on telomeres near the NE even in *Sun1-KO*,^13^ raising the possibility that TERB1 might be responsible for attachment plate formation, while SUN1 is, in part, required for its stabilization. This hypothesis is consistent with the preceding notions derived from studies in fission yeast and worms, in which the LINC complex is required for chromosome movement but is dispensable for proper attachment of the telomere/pairing center to the NE.^36^^,^^37^

Future studies, including electron microscopic observations of *Terb1-KO* telomeres or the identification of TERB1-associating membrane proteins, will further establish the molecular characteristics of the telomere attachment plate.
